# Becoming first time father of premature newborn during the first wave of the pandemic: a case study approach

**DOI:** 10.3389/fpsyg.2024.1391857

**Published:** 2024-07-26

**Authors:** Romuald Jean-Dit-Pannel, Chloé Dubroca, Flora Koliouli

**Affiliations:** ^1^Laboratory of Psychology, UR 3188, University of Franche-Comté, Besançon, France; ^2^Laboratory Psyche, School of Early Childhood Education, Faculty of Education, Aristotle University of Thessaloniki, Thessaloniki, Greece

**Keywords:** first-time fathers, prematurity, experienced separations, moments of connection, experiential approach, qualitative study, COVID-19

## Abstract

**Introduction:**

The aim of this paper is to delve into the emotional and psychological challenges that fathers face as they navigate the complexities of having a preterm infant in the NICU and in an unprecedented sanitary context.

**Methods:**

We used three data collection methods such as interviews (narrative and the Clinical Interview for Parents of High-risk Infants- CLIP) and the Edinburgh Postnatal Depression Scale (EPDS) to gain a comprehensive understanding of the cases.

**Results:**

The following analysis explores two individuals’ personal experiences of becoming a first-time father during the first wave of the COVID-19 pandemic through a close examination of two superordinate themes: “A series of separations through the experienced COVID- 19 restrictions” and “Moments of connection.” The transition to fatherhood is essentially with a medicalized form of connection with their newborn and the perceived paternal identity. In terms of temporality, these fathers experienced a combination of concerns about their infants’ long-term development and COVID-19 health concerns. Furthermore, they showed indications of phobic or hypochondriac tendencies using a psychoanalytic framework, along with an increased risk of postpartum depression.

## Introduction

Premature birth is a critical event that poses numerous challenges to newborns and their parents (Ionio et al., 2016). Existing research has largely focused on mothers’ experiences, but there is growing recognition of the importance of including fathers in studies on preterm infant care and development (Provenzi and Santoro, 2016; Filippa et al., 2021). Premature births can have profound psychological implications for fathers, often evoking feelings of helplessness and anxiety due to the suddenness and unpredictability of the event (Ionio et al., 2016; Koliouli et al., 2016a). Fathers may experience stress and fear about their infants’ health and survival, compounded by the unfamiliar and highly medicalized environment of the neonatal intensive care unit during the newborn’s hospitalization (Koliouli et al., 2016b; Stefana et al., 2018). However, the experienced stressed may persist several months after discharge (Salomè et al., 2022). Fathers of preterm infants may also struggle with the bonding process, as traditional interactions are disrupted by infants’ medical needs and the NICU setting (Mörelius et al., 2015; Koliouli et al., 2024). Feelings of exclusion from care decisions and pressure to provide emotional and financial support can further compound stress levels (Koliouli and Zaouche Gaudron, 2018). These psychological challenges can affect fathers’ mental health and relationship with the newborn, highlighting the need for targeted support and inclusion in the care of preterm infants (Koliouli et al., 2022, 2024).

### First-time fathers’ experiences

First-time fathers experience multiple psychological implications, as they encompass a wide range of emotional and functional changes as they transition into parenthood. This major life event can affect all aspects of one’s psychosocial functioning, with new fathers experiencing anxiety, which can vary significantly throughout their partner’s pregnancy and postnatal period (Philpott et al., 2019). Alterations in lifestyle and relationship dynamics also pose significant challenges, with fathers often feeling strain on their emotional and physical resources, which can have repercussions not only for their well-being but also for the partner’s and infant’s development (Hansson and Ahlborg, 2012). Adjusting to new responsibilities, sleep deprivation, and concerns about providing effective support can contribute to the stress experienced during this period. Further complicating this transition are changes in sexual relationships and the need for rest due to the demanding care a baby requires (Hansson and Ahlborg, 2012; Leavitt et al., 2017). Recognizing the complexity of these experiences, it is crucial to support first-time fathers in their new role of fostering positive outcomes for the entire family (Jean-Dit-Pannel et al., 2021).

Consequently, transition to fatherhood in the context of premature birth comes with a unique set of challenges as first-time fathers navigate the unexpected arrival of their newborn (Benzies and Magill-Evans, 2015). Their initial experience of fatherhood may be different from what they had anticipated as they grapple with the complexities of caring for a preterm newborn. From understanding the medical needs and potential complications associated with prematurity to emotionally processing the unexpected turn of events, first-time fathers find themselves in an uncharted territory. Furthermore, the COVID-19 context has further introduced uncertainties in this uncharted territory.

### COVID-19 context specificities

Transitioning to parenthood during the COVID-19 pandemic has added unique psychological challenges to first-time parents (Jean-Dit-Pannel, 2021). The combination of the typical stressors associated with the arrival of a new baby, such as adapting to new roles, altered sleeping patterns, and increased caregiving responsibilities, is further compounded by pandemic-related concerns. Transitioning to fatherhood during the COVID-19 pandemic has presented fathers with an array of challenges that distinguish their experiences from those in pre-pandemic times (Jean-Dit-Pannel and Belot, 2023). The health crisis has imposed limitations on fathers’ involvement in important prenatal events, such as attending ultrasounds or being present at their child’s birth, owing to hospital restrictions to limit the spread of the virus (Fonseca et al., 2023). Fathers, for example, have faced additional anxieties due to restrictions that might have prevented them from being present at prenatal appointments, delivery, and in the immediate postnatal period, significantly impacting their fatherhood experiences (Fonseca et al., 2023). The heightened risk of infection and its potential implications for the family’s health introduce an added layer of stress, which may have affected the quality of father-child interactions (Adama et al., 2022; Koliouli et al., 2024). Paternal parenting stress has been linked to higher anxiety levels during the pandemic, and this was particularly pronounced in areas with higher contagion rates, reflecting a direct impact on the father’s psychological well-being and the father-child relationship. This complex situation underscores the need for a responsive support system to assist new parents in managing these added stressors and to help safeguard their mental health during unprecedented times (Caporali et al., 2020; Adama et al., 2022). This has potentially hindered early opportunities to bond with their children and actively participate in the initial stages of caregiving. Moreover, expectant fathers might have had to experience the birth and early moments of their infant’s life via technological means rather than in person, altering the traditional first experiences with their newborns (Jean-Dit-Panell et al., 2024). The resulting impact on first-time fathers ranges from increased stress and anxiety to feelings of disenfranchisement in parenting roles. Despite these difficulties, some fathers have found new ways to engage by being more present at home, assisting with remote learning, and spending quality time fostering their children’s independence, resilience, and educational involvement (Fonseca et al., 2023). Such adaptations demonstrate the complex interplay between the challenging circumstances imposed by the pandemic and resilient responses that fatherhood can evoke.

Furthermore, the French NICUs varied in terms of imposed sanitary restrictions (Adama et al., 2022; Koliouli et al., 2024). However, they were mostly supportive about parents providing kangaroo care even during the COVID-19 period. Parents were involved in most caregiving practice, skin-to-skin contact included. It appears that the survival benefit of Kangaroo Mother Care far outweighs the small risk of death due to COVID-19. According to Minckas et al. (2021) the worst-case scenario of little probability (100% transmission in the NICU) could result in 1,950 neonatal deaths from COVID-19. Conversely, 125,680 neonatal lives could be saved with universal KMC coverage. In the same line, Kangaroo Father Care (KFC) may promote bonding of fathers with their infants and make them feel less stressed (Chavan et al., 2023).

To date, few studies have investigated the intricacies of becoming a first -time father of preterm infant, and this from a qualitative perspective (Benzies and Magill-Evans, 2015). To the best of knowledge, this is a first attempt to explore the singular context of the COVID-19 pandemic.

The aim of this paper is to delve into the emotional and psychological challenges that first-time fathers face as they navigate the complexities of having a preterm infant in the NICU and in an unprecedented sanitary context.

### Research design

Given the decrease in preterm births during the first wave of the pandemic in France (Fresson et al., 2022), we chose the case study approach to explore and better understand this phenomenon, that is the experiences of first-time fathers in a dually complex contexts: experiencing premature birth and the COVID-19 lockdown restrictions. These cumulative unique life conditions draw a unique context that a case study approach is suitable as method. We used the interpretative case study approach (Stake, 1995), which focuses on understanding the subjective and interpretative aspects of the research subject, often by delving deeply into selected cases. Interpretative case studies aim to grasp the meanings that participants attribute to their experiences and understand how those meanings influence their behavior. Finally, we undertook an instrumental case study (in terms of participants) which uses a particular case to gain a broader appreciation of an issue or phenomenon (Stake, 1995; Crowe et al., 2011).

## Methods

### Participants

Two fathers of premature newborns met in 10 months of the infants’ corrected age have participated to the study protocol.

Paul is 25 years old, he is a healthcare professional and has been in couple with his spouse for 10 years. Their baby boy was born in May 2020 at 34 weeks of amenorrhea (SA), a month and a half “too early.” This birth is medically qualified as “late prematurity.” Pregnancy occurred during the first lockdown. Due to this profession, he has been physically distanced from his pregnant spouse because of the risks linked to COVID-19 (“*not really knowing the risks there were with COVID-19 situation*”). Moreover, no warning signs of premature birth were observed: the pregnancy went well, and premature delivery had occurred a week after the couple’s reunion and was a surprise for both parents (“*we did not expect it at all*”). The duration of hospitalization was 1 month.

Franck is 31 years old, he is an Information Technology (IT) service manager in his branch. Franck and his spouse have confronted several challenges to conceive their child. Franck did not precise whether it was an IVF, but it took them 3 years to conceive. The pregnancy also occurred during the first lockdown and the couple did not experience any cumulative separations except for the time of birth. Their twin boys were born in June 2020 at 26 weeks of amenorrhea (SA), which is qualified as “extremely premature.” Franck was also the first parent who engaged in skin-to-skin practice (before the mother). They experienced 157 days of hospitalization in hospital and 2 months of neonatal care at home (providing home medical and paramedical care depending on the evolution of the baby’s health).^1^

The experienced restrictions that these NICUs imposed were as per the extended family visitations: parents could practice skin-to-skin freely and get involved in caregiving practices.

### Data collection

We used three data collection methods such as interviews (an interview narrative and the Clinical Interview for parents of High-risk infants) and the Edinburgh Postnatal Depression Scale to gain a comprehensive understanding of the case.

### Narrative interview

The unstructured interviews began with a simple question, designed to be as broad as possible to encourage a free discussion: “Would you like to tell me about your transition to fatherhood, your experience of becoming a father of a premature newborn?” We deliberately chose to stress the experience of becoming a father before invoking the COVID-19 pandemic. Following the non-directive nature of the narrative interviews, we respected the silences of the participants, and the prompt questions were used to further develop their narratives (i.e., “*Could you please further explain? Could you give us an example? “What do you mean by this*?”).

### Clinical interview for parents of high-risk infants

The Clinical Interview for Parents of High-risk Infants (CLIP) (Meyer et al., 1993) is a semi-structured interview that explores the relationship of parents with the healthcare team on the one hand and with the family on the other hand, and the provided social support. In our study, we used a version adapted for parents of premature babies (Meyer et al., 1993). This tool can detect both the psychological situation of the parents (their needs, fears, etc.) and can also constitute a means of managing these difficult experiences for the parents. In other words, parents can express themselves through open questions about their experiences in the neonatology unit. This semi-structured interview explored the following axes:

- Infant’s current condition: further explore how the father perceives his baby’s condition. The description of the baby’s condition aims to introduce a more emotional aspect and goes beyond a simple collection of medical information, namely possible respiratory problems, breastfeeding etc.
- Course of pregnancy: The description of the pregnancy course depends on the sensitivity of the father towards his partner, but also on his involvement from the beginning of the pregnancy.
- Labor and delivery: This section refers to the presence of the father during labor and whether he was the first to meet the newborn.
- Relationship with the baby and feelings as a parent: This section covers the first paternal feelings when first meeting his baby.
- Reaction to the Intensive Care Nursery and Relationships with Staff: The purpose of this group of questions covers reactions towards the medical equipment of the unit and its relationships with healthcare. Thus, fathers were asked to identify the sources of perceived stress and the most supportive persons around them.
- Relationship with Family and Social Support: Explore the perceived support sources among family and friends.
- Discharge and beyond: In the section, we tried to explore paternal reflections about the future through projections as a father.

### Edinburgh postnatal depression scale

The Edinburgh Postnatal Depression Scale (EPDS), originally developed by Cox et al. (1987), contains 10 items with four response options, each rated 0–3. EPDS is a self-administered screening tool that is used to evaluate postpartum depression. Thus, the range is 0–30 points, with higher scores indicating more depressive symptoms. The EPDS asks if, during the last 7 days, the respondent had been able to laugh/see the funny side of things, looked forward to things with enjoyment, blamed oneself unnecessarily when things went wrong, been anxious or worried for no good reason, felt scared or panicky for no good reason, felt that things had been “getting on top” [of the respondent], had been so unhappy that it had been difficult to sleep, had felt sad or miserable, had been so unhappy that [the respondent] had been crying, and if the thought of harming oneself had occurred to the respondent. The EPDS has also been validated for fathers by Matthey et al. (2001), who demonstrated that a score of 9/10 or more was optimal for the detection of minor and/or major depression, with a sensitivity of 71.4%, a specificity of 93.8%, and a positive predictive value of 29.4% (Carlberg et al., 2018). We used the French validation by Guedeney and Fermanian (1998) and Adouard et al. (2005).

### Procedure

Fathers were invited via websites and social media platforms and through the SOS Préma support organization for NICU parents in France. We expected to recruit more fathers, however solely two fathers accepted to participate.

The study protocol (interviews and EPDS) was conducted when every newborn was at 10 months of corrected age. The whole procedure was recorded for transcription, and the duration was approximatively 1 h and a half. Participants responded firstly at the narrative interview and then at the CLIP. Finally, they completed the EPDS. It is important to note that the interviews conducted in French were translated into English by the third author after the analysis and then verified by the other two authors. As noted by Temple and Young (2004), the act of translation must be acknowledged, as the researcher’s sociocultural positioning is inextricably linked to the interpretation of the findings.

### Data analysis

An interpretative phenomenological approach was employed with the data analyzed as follows (Smith et al., 1999). Initially, the transcripts were read and re-read several times to obtain a holistic picture of each participant’s narrative. During this process, the first researcher made unfocused notes related to anything within the text that appeared interesting or significant. Following this, the transcripts were examined further, and conceptual themes were created to capture the essence of the participant’s account. The emergent themes were listed, and connected themes were sought, with those related to being clustered under appropriate superordinate conceptual headings. During this data organization process, all three researchers continuously referred to the transcripts to ensure that the themes selected were representative of the participant’s accounts. Themes should reflect the most salient meanings within the participants’ narratives. The resultant framework, consisting of emergent experiential and psychological clusters, was used to facilitate the production of the written analysis. The iterative process continued throughout the analysis to ensure that the data were appropriately represented. In addition, an independent ‘auditor’ checked whether interpretations are warranted against the data. These validation criteria were adhered to in this study.

We used a phenomenological approach to focus on the lived experiences, so we followed an inductive data analysis method. However, an essential component of methodological rigor in qualitative analysis is reflexivity (Malterud, 2001) in order to being aware of our own position in producing research. In this study, all three researchers are trained psychologists in psychoanalytic/psychodynamic approaches. The study protocol was done by one researcher, so we acknowledge that collected data are dependent on the interviewer’s questions and prompts (Sale, 2022). Furthermore, the data conceptualization may have been influenced by this clinical analytic process.

### Ethical considerations

The study was conducted in accordance with the local legislation and institutional requirements (Department of Psychology of the University of Franche-Comté, in France). The participants provided their written informed consent to participate in this study, before the data collection procedure started. Due to the constraints posed by the ongoing pandemic, researchers secured digital letters of permission with affixed e-signatures from the participants. The participants were further verbally asked if they consented to be interviewed during the scheduled interview date. Participants were encouraged to narrate their experiences freely and honestly in response to questions. They were also given the discretion to refuse to answer a question or cease participating at any time if they wished to do so. Furthermore, considering the sensitive nature of this research, the participants were allowed to contact a trained psychologist who conducted the interviews. Finally, all names were pseudonymized to safeguard participants’ anonymity.

### Findings

The following analysis explores two individuals’ personal experiences of becoming a first-time father during the first wave of the COVID-19 pandemic through a close examination of two superordinate themes. The first one is named “A series of separations through the experienced COVID- 19 restrictions,” and the second one “Moments of connection.” The first theme details the restrictions experienced on different levels of transition to fatherhood, and the second delves into the moments that connected fathers with their experienced fatherhood and the lasting latent psychoemotional implications of this period. For ease of explanation, the two themes were presented separately, although there were several interrelated elements.

### A series of separations through the experienced COVID-19 restrictions

#### Experienced separation during pregnancy and psychoemotional reactions

Paul largely expressed that the pandemic had a strong impact on the course of the pregnancy because he was not able to experience it with his wife fully. More specifically, Paul claims that the fact of not being physically with his wife and not observing her body changing has complexified his transition to being a father.

“*We spent a lot of time alone, away from each other, and I spent a lot of time of this pregnancy alone too, so we didn't see each other much and it was after the end of this first lockdown when she came back. So, we did not really enjoy the pregnancy together... She actually went into labor a week after she had returned to our home*.” Paul explained that his spouse was off to the parents’ house to protect herself and the future infant from the COVID-19 risks “*Euh, so uh not knowing too much about the risks involved with covid in fact she, she went back to live with her parents in fact during the period when I was working.”*

Paul pointed out that he felt excluded from the course of pregnancy because of his profession (being confronted day-to-day with the COVID-19 virus) that prevented him to fully experience it. Despite his work status (that is being a healthcare professional), Paul experienced feelings of helplessness and frustration as he could not be actively involved during his spouse’s pregnancy in terms of prenatal screenings and preparations.

“*The fact of experiencing this pregnancy from afar, without being able to participate, and without having the impression that I was actively involved in this moment, it was complicated. I mean, I do have any memories of it, because I was not there* (laughs)!”

The COVID-19 context seems to have played an important role in Paul’s narrative. He graphically expressed that the sanitary crisis had a strong impact on this pregnancy, as he was not able to fully experience it with his wife. He also wondered whether this context was not a potential cause of prematurity given the perceived stress during that period: “*Would the pregnancy have gone more smoothly, would it have gone further, would it have gone better, would things have been different? Was it the overall stress of pregnancy related to this covid period that was complicated? we’ll never know! It certainly leaves a little taste of unfinished business (laughs).”*

Franck was neither present at prenatal screening. His narrative differs from that of the previous one. At the first appointment, their doctor (gynecologist) was 3 h later; Franck was unable to stay as he had to return to work, thinking that he would be able to attend many more ultrasound examinations afterward. Unfortunately, strict restriction measures were applied, and he was no longer able to attend. The healthcare professionals sent video recaps of the ultrasounds and prenatal screening, which further enhanced his experience of disconnection.

“*Small disappointment this COVID-19 situation [meaning in the way that he experienced his spouse’s pregnancy] because it was the first lockdown which means that it was not to join the mom to the various exams, ultrasound. In fact, strangely enough, it's frustration I am feeling today because at the time I was saying to myself: "Well, as long as everything's going well*….”

At 22 SA, the future parents rushed to the emergency room when one of the amniotic sacs ruptured. Unfortunately, Franck was unable to join his spouse because of hospital restrictions. Franck’s spouse remained in the hospital for 5 days without any authorized visitors, including Franck.

“*Uh so there too uh moment of fear and frustration because I'm dropping my wife off at the emergency entrances […] I couldn’t go with her…. The restriction would not let me join her, so she stays, she and the babies for five days in the high-risk pregnancy section, with no visits, no way to see each other, no way to comfort her, and no way to support her. uh, so that was the first shock of having to abandon her to ... and seeing her from the forecourt, outside the hospital.”*

In his narratives, Franck unraveled fear, a sense of helplessness, and frustration. Indeed, several separations and disconnections before labor and delivery are noted: for instance, the 3 years to conceive, the 5 days of high-risk pregnancy hospitalization… “*there was no support or soever to be with my spouse for prenatal screening and examinations*,” “*ultrasounds*,” or even being able to “*attend childbirth preparation courses*.”

### Newborn’s hospitalization and suspended paternal identity

Paul’s son spent a short time in the intensive care unit and then joined the neonatal unit. He stayed there for a month and a half. During this time, Paul found it difficult to assume his new identity as father. The constant uncertainty, coupled with anxiety and the stressful experiences of the labor, here experienced as a traumatic event, also made this entry into fatherhood complicated.

"*So, it's clear that for the first few weeks I did not really... it was complicated to get it into my head that I was a dad and that Simon had arrived.*"

As for Franck, he reminisces, relieved, the first night when his boys were born: “*during the night, it was like they tried [healthcare team] to make room for the boys*,” thus providing additional space to accommodate their twins. Two hours later, they were invited to visit their children to the pediatric intensive care unit. They were shocked and stayed only 10 min: “*We’re in for a phenomenal slap in the face*.” The medical equipment (screens, syringes, and tubes) around his boys were unbearable to both Franck and his spouse.

"*We see our little ones, who don't look like babies as you might imagine... They look red and are intubated, probed, and monitored. They are no more than 30 centimeters long, er... 800 grams*". Franck’s wording illustrates that he has been traumatized by the medical environment.

He also pointed out that he would feel his “first feeling as a father,” as he described it: “*my first paternal emotion, well, I felt like a day after their birth*.” Moreover, Franck shared his first reflections on realizing that he was a father. Despite the challenges and dark thoughts that may have occurred, he was motivated to be closely present to his twins. “*And then I said to myself ... you are a dad, you have got your boys, you have got to go. And then I go back to see the kids and uh, pretty dark thoughts. (nervous laughter) I have to take care of them.”* He became emotional when he verbalized the worst scenario of his newborns’ hospitalization: “*If they live 3–4 days, then even for 3–4 days they need a dad. Erm...so I stay there, I do not know how long either, but a long, long time*.”

It seems that paternal identity in the context of premature birth is somewhat suspended: the medicalized context impedes their transition to fatherhood because they are primarily focused on newborns’ health issues, survival, and well-being. This results in a paternal preoccupation that prevents them from fully experiencing their paternal identity, as if it is on pause, waiting to see if their babies will survive. “*Looking back now, what we have been through, it is very emotional, you know*” (Franck).

### Moments of connection

#### Labor and delivery: questions about life and death

Paul expressed his vivid memories of the moments before his spouse’s labor. Firstly, he recalled the moment when the “water broke” and had to drive his spouse to the hospital. He was in complete confusion and uncertainty, stating that he was afraid for his son’s life. He relates that his anxiety was linked to the uncertainty of the baby’s state of health and that it was still “too early” for him to be born.

“*So, it's still something magical... and at the same time it's a huge stress because we don't know: is he going to have after-effects uh is he uh going to survive uh is he going to be okay, is he uh and the problem is that until he arrives and until he's been examined uh we can't know... Uh so uh that, so yes we'll say that a moment, it's a fuzzy moment where we don't really know where we are, emotionally we're neither happy nor not happy uh and here we are navigating we're navigating between these two states constantly*”

Paul was present in the delivery room thanks to a midwife who decided to let him in. He had anticipated not being able to attend, so his presence was experienced as a chance, even a privilege. Despite Paul being a healthcare professional, he was very worried and stressed after the birth of his son because he manifested pulmonary difficulties.

Franck had also attended at his children’s birth. He described it as “*anxious*” due to the presence of numerous resuscitators. Their newborns were intubated directly, and Franck stated that “*This is not how you imagine the birth of your child.*” He described in detail the delivery process that was stressful and challenging as their newborns were resuscitated.

“*Well, between the birth of L and T… On one side, I could see my wife preparing to give birth to T, while on the other side, in a separate room with an open door, I could see them giving L his initial treatments. However, these were not his first treatments, as the initial care should involve cleaning and aspiration of the nose. We cleaned him up and provided the necessary resuscitation care. And, trust me, nobody is prepared for this*…”

It seems that both fathers experienced the delivery process as both magical and stressful at the same time. On one hand, there’s a sense of awe and wonder, but on the other hand, there’s uncertainty and anxiety about whether the newborns will experience any negative consequences, whether they will survive, and whether they will be healthy. The uncertainty that resides did not let these fathers to fully relate to the positive emotions.

#### Skin-to-skin and caregiving practices: fighting against detachment

Paul described that he followed his son in the NICU and he started skin-to-skin practice since day one. Paul felt that the first bond was formed through the physical contact and interaction. However, he encountered challenges in finding his place as father and played an active role in the caregiving process.

“*It is true that we were lucky to be able to do skin-to-skin very quickly […] So, uh that was really good, it allowed us to have a real sharing contact with him right away. It is true that the nurses and pediatricians at the hospital in X (name of the town) were very supportive*.”

However, Paul experienced feelings of dispossession due to the continuous and invasive presence of both the medical staff and equipment around his son.

“*During the ICU period, uh, after neonatal care less so, but on the other hand, it is true that in the ICU period, yes, there is so much, there is so much equipment around. […] There are so many people constantly coming to look after him that it's almost as if we are doing nothing, apart from skin-to-skin contact and trying to feed him a little from time to time when they suggest it, the rest of the time we feel like we are doing nothing. It's like being a spectator and waiting for things to unravel*”

Skin-to-skin contact and feeding the newborn were among the caregiving activities that initiated these new fathers into parenthood. Franck also participated in his child’s first bath, as he described, although they had their “firsts” in the NICU, it was under exceptional conditions. “I *fed my son his first bottle using a syringe, but it was still my first bottle (smiling). The first bath was also under special conditions because he could not be separated from his breathing machine for too long, which is normal*.” Franck was involved in the caregiving process, and he felt that it made him truly feel like a father. The first experiences of these fathers have a unique quality because there are several safety precautions to consider that they can cause anxiety and apprehension, enhancing the “medicalized” sense of fatherhood.

Franck also shared his experiences with the supportive role of healthcare professionals in caregiving. For instance, he was not feeling capable of having skin-to-skin on his own. Thus, the first physical contact between Franck and his twins was enabled by the healthcare staff. He described vividly that “*they did not have any reaction at first […] you have to imagine, they were under morphine […] They must be physically shocked too*.” Franck exhibited active empathy towards his newborns, talking more about how they would feel instead of himself, thus enhancing his connection to them.

Nevertheless, he expressed feelings of guilt without specifying whether they were about his partner and/or twins. “*I had to stay 3–4 h with the boys and then I say to myself, I have to go check on the mom too... Since I cannot find her on the phone at all, I must go and check her. So, here we were, returning to the NICU with their mother, well it’s just as complicated, but uh... It is even more complicated because of guilt*.”

In his narratives, we witnessed a conflict that involved an internal struggle between Franck’s real and imagined worries regarding his spouse and their twins, which had an impact on the quantity and quality of their interactions with the baby. Despite the moments of connection experienced during caregiving practices, Franck also tried to connect his twins with their mothers, build bridges, and fight against disconnections and disruptions (Gaudron et al., 2016).

“*[A piece of advice for parents] is to spend time with their child, to create a different bond, but to create it quickly, through skin-to-skin contact, caresses, caregiving, [in prematurity] it's not an encounter as we may have in mind, but it's still an encounter with our child*.”

#### Reflecting on perinatal experiences: the importance of temporality

In terms of temporality, we explore the changes in their experiences over time and in relation to the dynamic relationship between fathers, their infants, and spouses. Both fathers have completed the self-administered EPDS scale at 1012 months of corrected age of their newborns. Paul obtained 13 and rated very high the item that “he very often felt worried or worried without reason.” Franck, on the other hand, had obtained 12 (see Table 1). These scores indicate an increased risk for postnatal depression and anxiety for up to approximately one year following the premature birth.

**Table 1:** Description of EPDS scores.

Items			*Paul’s scores*	*Franck’s scores*
1. I have been able to laugh and see the funny side of things:	As much as I always could	0		
	Not quite so much now	1	1	
	Definitely not so much now	2		2
	Not at all	3		
2. I have looked forward with enjoyment to things:	As much as I ever did	0		
	Rather less than I used to	1	1	1
	Definitely less than I used to	2		
	Hardly at all	3		
3. I have blamed myself unnecessarily when things went wrong:	No, never	0		0
	Not very often	1		
	Yes, some of the time	2	2	
	Yes, most of the time	3		
4. I have been anxious or worried for no good reason	No, not at all	0		
	Hardly ever	1		
	Yes, sometimes	2		2
	Yes, very often	3	3	
5. I have felt scared or panicky for no good reason	Yes, quite a lot	3		
	Yes, sometimes	2	2	
	No, not much	1		1
	No, not at all	0		
6. Things have been getting to me	Yes, most of the time I have not been able to cope at all	3		
	Yes, sometimes I have not been coping as well as usual	2		
	No, most of the time I have coped quite well	1	1	1
	No, I have been coping as well as ever	0		
7. I have been so unhappy that I have had difficulty sleeping	Yes, most of the time	3		
	Yes, sometimes	2	2	2
	No, not very often	1		
	No, not at all	0		
8. I have felt sad or miserable	Yes, most of the time	3		
	Yes, quite often	2		2
	Not very often	1	1	
	No, not at all	0		
9. I have been so unhappy that I have been crying	Yes, most of the time	3		
	Yes, quite often	2		
	Only occasionally	1		1
	No, never	0	0	
10. The thought of harming myself has occurred to me	Yes, quite often	3		
	Sometimes	2		
	Hardly ever	1		
	Never	0	0	0
**Total score**			13	12

Reflecting on their perinatal experiences, Paul felt that it was no longer possible to state whether his son was born prematurely, meaning that his development was typical. However, he confided that he was still worried about his son’s state of health and the fear of an eventual disability that has not been still manifested, even though he is currently developing well.

“*It’s difficult to remain calm… Every beginning, everything has been difficult and challenging so far… […] We are still worried about him […] maybe because of his fragility [being born prematurely]”*

During the interviews, these fears and worries about his son’s long-term development are merged with the COVID-19 health worries. We might hypothesize that Paul encountered “psychoemotional residuals” in terms of obsessive, phobic, and hypochondriacal tendencies, translated by his fear of contamination, but also his fear of illness at the slightest sign in his child.

“*Because of being born prematurely, so now every time who has anything I see that he is a little sick every time, we always put a brake on ourselves and we doubt and we say to ourselves is it going to be okay is it not serious is this and.. we think back to all these moments and we say to ourselves ah pff I hope it's not okay, it's not going to get worse. So, there's already that, I think it's difficult, it must not be difficult for parents of non-premature children, but having a premie, I think it brings even more anxiety*”

In the same line, Franck conveyed his concerns about the developmental outcomes and the emotional burden he had experienced. More specifically, the prospect of having a child with developmental delays or disabilities is a topic that really worried Franck.

“*We know that we will have an impact on T but that we will not have a serious handicap. The possibility of having a child with a delay or a handicap that… it's something that's coming back strongly now*.”

In his case, both twins had experienced hemorrhages that could possibly have an impact in their future development. Franck’s worries are linked to the current health condition of his two infants. “L*, has fully reabsorbed the hemorrhage, while T had two hemorrhages in distinct locations. T has successfully reabsorbed the first hemorrhage, but still has small lesions in the parenchyma. Compared to the first images, which showed significant reabsorption, the reabsorption of the first hemorrhage is still very well-managed*.” Franck has used a quite medical terminology to fully explain his infants’ health condition, which is common among fathers of premature infants (Koliouli et al., 2016a). “*According to the current outlook, it will be approximately 2–3 years before we see any progress. The condition known as leukomalacia [that T has been diagnosed with] is constantly on my mind, and I must think about it numerous times each day*.”

The fathers, having discussed the challenges their infants may face in the future, made a concerted effort to focus on the positive aspects of their development and to be more attentive to their current needs.

“*He does so many things that, it's, it's a wonder every day, even if at times afterwards it's a lot of anxiety or sometimes for other things but it's still quite magical*” (Paul)

"*Well now they're progressing at their own pace, at different rates for each other, but… they're making progress. At that moment, well, they are in the top form (smiles)…! […] both our children are miracles, according to the doctors*." (Franck)

### Descriptive analysis of the EPDS

Paul obtained a total score of 13 when he completed this self-questionnaire 10 months and 10 days after the premature birth of his child born on May 31, 2020, during the covid-19 health crisis, at 34 weeks’ amenorrhea.

Franck’s scores on the EPDS scale were 12 when he completed this self-questionnaire 10 months and 11 days after the premature birth of his twins, born on June 30, 2020, during the covid-19 health crisis, at 26 weeks of amenorrhea (see Table 1).

We can observe four common responses from both fathers (Items 2, 6, 7, and 10):

- These fathers felt less confident and joyful than usual when thinking about their futures (Item 2).
- They were able to cope with most of the events (itemtem.6);
- Sometimes, they felt so unhappy that they had trouble sleeping (Item7);
- They never thought of hurting them. (Item 10).

There was only one rating of three for Paul (4-I felt worried or concerned for no reason: yes, very often (3)).

## Discussion

The aim of this study was to explore the experienced psycho-emotional challenges first-time fathers face as they navigate the complexities of having a preterm infant in the NICU and during an unprecedented sanitary context by using interpretative case study approach. The major themes that were identified offer an insight in the experiences of first-time fathers during the pandemic which were full of separations and moments of connection. The first theme is focused on the series of separations through the experienced COVID- 19 restrictions, and the second one on the “Moments of connection.” However, when taking into account the temporality dimension, the different subthemes are intrinsically interrelated one to another.

More specifically, the two fathers identified several events of separations and disconnections during pregnancy which generated dysphoric emotions. For Paul, separation during his spouse’s pregnancy was devastating, as he was unable to be physically present during prenatal appointments and other important milestones. COVID-19 restrictions further exacerbated his feelings of helplessness and isolation. Franck also experienced a profound sense of loss and disconnection due to the restrictions. He had only had the experience of a virtual view of his spouse’s pregnancy, which, in our opinion, amplified the virtual nature of this pregnancy and his difficulty in materializing her children. Paternal experienced exclusion produces feelings of isolation, and a sense of loss, along with a disconnection from the pregnancy, is also found in existing research (Andrews et al., 2022; Poulos et al., 2022).

The medical context prioritizes the newborn’s health, survival, and well-being and may divert the attention of fathers from their transition into fatherhood. Both fathers highlighted their constant uncertainty, intense worry, and challenges in interacting with newborns. These narratives align with previous research findings with larger sample sizes (Lasiuk et al., 2013; Koliouli et al., 2016a). Additionally, we uncovered fathers’ nuanced concerns, preoccupations, and dark thoughts surrounding their newborns’ hospitalization. It is important to recognize that when infants are hospitalized, fathers are unable to fully embrace their roles or establish their own new identity.

This series of separations have introduced additional challenges in the construction of their primipaternal identity, as they often oscillated between feelings of joy and intense worry. These fathers were initially worried about their newborns’ survival and had experienced a brutal delivery process followed by their infants’ long-term hospitalization. Thus, their primipaternal identity during this important phase of their transition to fatherhood is intrinsically associated with a medicalized form of connection with their newborn. This suggests a novel aspect of paternal experience in the context of premature births, as fathers are largely preoccupied by the medical assistance and follow-ups that their newborns need and, at the same time, consist of an essential part of their relationship with their newborn. In existing research, first-time fathers’ experiences have mostly been viewed in the context of full-term births (Fonseca et al., 2023), not considering the additional disruptions and sources of anxiety that a premature birth holds.

The second theme, “Moments of connection,” highlighted the importance of small interactions and bonding opportunities between fathers and preterm infants in the NICU. For both Paul and Franck, labor and delivery were the first moments of connection with their children. In the current literature, paternal presence during labor and delivery empowers their paternal role, increases positive feelings about fatherhood and birth, and improves maternal attitudes towards the father (Sapountzi-Krepia et al., 2015; Smith et al., 2024). However, in the context of premature birth, feelings of joy were mixed with increased stress and anxiety as our participants’ newborns were transferred to intensive care. Despite the stressful and traumatic aspect, it seems that early paternal presence empowered Paul and Franck, as they could actively support their spouse and share this unique experience after a series of separations.

Skin-to-skin contact was experienced as an active way to bond and connect with their child during the restrictive measures that separated them during pregnancy. These moments provided a sense of purpose and connection for both fathers, allowing them to feel involved in their child’s care and development despite challenging circumstances. Although there is little research on paternal skin-to-skin in the COVID-19 context, the existing literature suggests that paternal skin-to-skin reduces anxiety and depression in fathers and facilitates their role attainment (Olsson et al., 2017; Huang et al., 2019; Koliouli et al., 2022). However, due to the COVID-19 pandemic and the associated restrictions in hospitals, parents have faced challenges in engaging with skin-to-skin (Saus-Ortega, 2023), affecting their emotional well-being (Adama et al., 2022). It seems however that in the French NICU where the fathers of our sample had their newborns hospitalized acknowledged the importance of the kangaroo care even in pandemic times (Minckas et al., 2021).

Intersubjective temporality refers to the shared understanding of time between individuals in a social context (Rodemeyer, 2006). Through this shared comprehension of time, people are able to align their actions and perceptions within a group or community. We assumed that this concept can be applied to the transition to fatherhood by exploring how a father’s experiences and perceptions about their newborn evolves as he assumes the role of the caregiver. In this case study, it is translated through the growing presence of fathers in the caregiving process. The concept of “psycho-emotional residuals” encompasses persistent and enduring anxieties and apprehensions that originate during childbirth and continue to evolve in accordance with the health status of the infant. Drawing on our knowledge of psychoemotional residuals, we might surmise that both fathers grappled with obsessive, phobic, and hypochondriacal tendencies, which manifested in fear of contamination and anxiety over their infants’ health conditions (Kernberg, 2004). In a psychoanalytic approach, obsessive, phobic, and hypochondriacal tendencies are often linked to underlying psychological conflicts and unresolved issues (Kernberg, 2004). These tendencies can be seen as defense mechanisms that individuals use to cope with unconscious anxiety and fear. Fon instance, a person with hypochondriacal tendencies may constantly worry about their health and seek reassurance from others because of underlying fears of illness or death (Nissen, 2018). Moreover, these traits were accompanied by the risk of increased symptoms of post-partum depression, as the EPDS scores suggest. Paul and Franck obtained high scores that indicate an early onset of intense worry, anxiety and possible depressive symptoms. Paternal early post-partum depression signs are often overlooked during the perinatal period (Eddy et al., 2019; Wang et al., 2021), particularly in the context of premature birth.

## Limitations

Our study focused on participants’ subjective experiences during the first antenatal period to understand them at a specific time and place. The data collection was conducted on one session with the fathers. Thus, it would be interesting to explore lived experiences from a longitudinal perspective, and further systematic, prospective analysis on paternal lived experiences is needed. Furthermore, some methodological concerns may be pointed out: the small sample of two fathers may be restrictive and we did expect to have more participants enrolled. Nevertheless, in a case study approach, the focus is on the phenomenon rather than the number of participants (Stake, 1995; Crowe et al., 2011). Moreover, we acknowledge that a case study can largely rely on the researcher’s interpretation and selection of data, thus contributing to the “researcher’s bias.” Nonetheless, we managed data triangulation through different sets of data collection and ensured overall supervision by experienced researchers. Another limitation might be the exclusive focus of the paternal perspective on their experiences of psycho-emotional challenges in becoming first-time fathers of premature newborns during the pandemic period. Given that meaning and experiences are co-constructed (Smith et al., 1999), it is worth considering the perspectives and lived experiences of the father’s spouse and extended family as active participants in the construction of fatherhood experience. Last but not least, EPDS is a very sensitive questionnaire and results could be influenced by the moment of administration. Furthermore, accumulating research evidence shows that EPDS might not be the best suited tool to assess the risk of depression in fathers (Matthey et al., 2001; Baldoni and Giannotti, 2020; Walsh et al., 2020) and different set of self-reported questionnaires should be used to better assess the early onset signs of anxiety and/or depression among fathers.

## Future research and implications for practice

Transitioning to fatherhood and fathers’ mental health in perinatal settings, including premature birth, is underrepresented in research. Therefore, further research should be conducted on these subjects, and future research should focus on issues of grief and loss in perinatal settings and on the importance of moments of connection.

Our study also highlights the importance of early involvement in caregiving processes for fathers, to enable their experienced transition to fatherhood, especially in the context of premature birth. Skin-to-skin contact (which has been explored in existing literature) was practiced during the pandemic period and, as highlighted in our results, provided a sense of connection to the newborn and to the new role as a father. Thus, NICUs should consider undisrupted involvement in caregiving during premature birth, which further enhances the early bonding between the father and newborn.

From a preventive perspective, the EPDS scale is a simple tool that can provide healthcare professionals with information about the risk of anxiety and depression symptoms in NICU parents.

## Conclusion

It is important to understand the fathers’ unique experiences and the need to provide appropriate support during this challenging period. This case study contributes to the existing literature by exploring the range of experiences of fathers with preterm infants in the neonatal intensive care unit and during their transition to home. It examines psychosocial implications, such as stress and anxiety related to a fragile newborn and the uncertainty of the infant’s health and development. Furthermore, this study extends its focus to the transition period when the preterm infant is discharged from the NICU and the family returns home. It delves into the challenges that fathers encounter during this transition, such as adjusting to new caregiving responsibilities, managing the financial burden of medical expenses, and coping with the infant’s ongoing medical needs. By shedding light on the multifaceted experiences of fathers with preterm infants, this case study sought to provide valuable insights for healthcare professionals, policymakers, and support organizations to develop more comprehensive and effective support systems for fathers in similar situations.

This research not only highlights the importance of including fathers in the discourse on preterm infant care but also advocates for tailored interventions that address the specific needs of fathers during this challenging period. Specifically, recommendations are provided to enhance the involvement of fathers in the NICU and at home, emphasizing the promotion of father-infant relationships (Baldoni et al., 2021). These recommendations address the need for health care professionals to acknowledge and support fathers’ emotional well-being, provide education and resources on infant care, involve fathers in decision-making processes, and establish support networks for fathers of preterm infants (Stefana et al., 2022; Bréhat et al., 2024).

## Data availability statement

The raw data supporting the conclusions of this article will be made available by the authors, without undue reservation.

## Ethics statement

Written informed consent was obtained from the individual(s) for the publication of any potentially identifiable images or data included in this article.

## Author contributions

RJ-D-P: Conceptualization, Data curation, Formal analysis, Funding acquisition, Investigation, Methodology, Project administration, Resources, Software, Supervision, Validation, Visualization, Writing – original draft, Writing – review & editing. CD: Conceptualization, Investigation, Methodology, Visualization, Writing – original draft. FK: Conceptualization, Formal analysis, Funding acquisition, Methodology, Resources, Supervision, Validation, Visualization, Writing – review & editing.

## Footnotes

^1^In France, neonatal home care has advanced in response to familial demands and is part of the evolution of neonatology towards family-centered care. Infants who are not yet mature and need neonatal care, like feeding through tubes, can continue to receive hospital care at home, with the help of specialized teams in collaboration with a neonatology unit (Montjaux-Régis et al., 2023).
